# Using Artificial Intelligence to Detect Risk of Family Violence: Protocol for a Systematic Review and Meta-Analysis

**DOI:** 10.2196/54966

**Published:** 2024-12-02

**Authors:** Kathleen de Boer, Jessica L Mackelprang, Maja Nedeljkovic, Denny Meyer, Ravi Iyer

**Affiliations:** 1 Department of Psychological Sciences Swinburne University of Technology Hawthorn Australia

**Keywords:** family violence, artificial intelligence, natural language processing, voice signal characteristics, public health, behaviors, research literature, policy, prevalence, detection, social policy, prevention, machine learning, mental health, suicide risk, psychological distress

## Abstract

**Background:**

Despite the implementation of prevention strategies, family violence continues to be a prevalent issue worldwide. Current strategies to reduce family violence have demonstrated mixed success and innovative approaches are needed urgently to prevent the occurrence of family violence. Incorporating artificial intelligence (AI) into prevention strategies is gaining research attention, particularly the use of textual or voice signal data to detect individuals at risk of perpetrating family violence. However, no review to date has collated extant research regarding how accurate AI is at identifying individuals who are at risk of perpetrating family violence.

**Objective:**

The primary aim of this systematic review and meta-analysis is to assess the accuracy of AI models in differentiating between individuals at risk of engaging in family violence versus those who are not using textual or voice signal data.

**Methods:**

The following databases will be searched from conception to the search date: IEEE Xplore, PubMed, PsycINFO, EBSCOhost (Psychology and Behavioral Sciences collection), and Computers and Applied Sciences Complete. ProQuest Dissertations and Theses A&I will also be used to search the grey literature. Studies will be included if they report on human adults and use machine learning to differentiate between low and high risk of family violence perpetration. Studies may use voice signal data or linguistic (textual) data and must report levels of accuracy in determining risk. In the data screening and full-text review and quality analysis phases, 2 researchers will review the search results and discrepancies and decisions will be resolved through masked review of a third researcher. Results will be reported in a narrative synthesis. In addition, a random effects meta-analysis will be conducted using the area under the receiver operating curve reported in the included studies, assuming sufficient eligible studies are identified. Methodological quality of included studies will be assessed using the risk of bias tool in nonrandomized studies of interventions.

**Results:**

As of October 2024, the search has not commenced. The review will document the state of the research concerning the accuracy of AI models in detecting the risk of family violence perpetration using textual or voice signal data. Results will be presented in the form of a narrative synthesis. Results of the meta-analysis will be summarized in tabular form and using a forest plot.

**Conclusions:**

The findings from this study will clarify the state of the literature on the accuracy of machine learning models to identify individuals who are at high risk of perpetuating family violence. Findings may be used to inform the development of AI and machine learning models that can be used to support possible prevention strategies.

**Trial Registration:**

PROSPERO CRD42023481174; https://www.crd.york.ac.uk/prospero/display_record.php?RecordID=481174

**International Registered Report Identifier (IRRID):**

PRR1-10.2196/54966

## Introduction

Family violence is a major public health problem and can include behaviors such as physical, sexual, and verbal abuse; coercive control; and emotional, spiritual, religious, and financial abuse perpetrated by 1 adult partner to another. Definitions of family violence vary in the research literature, which has implications for reported rates of family violence and, importantly, policy [1]. For this article, family violence will be defined by Section 4AB (1) of the Australian Family Law Act 1975 as “threatening or other behavior by a person that coerces or controls a member of the person’s family (the family member) or causes the family member to be fearful.” Family violence is most commonly, but not exclusively, perpetrated by men against women [2], and these experiences can have considerable mental health impacts, such as depression, posttraumatic stress disorder and anxiety [3], and physical consequences for survivors, including serious injury and death [4]. Recent estimates suggest that globally 27% of women aged between 15 and 49 years old have experienced intimate partner violence [5]. In Australia, the most recent statistics suggest that approximately 20% of adults have experienced family violence at some point since the age of 15 years [6].

The prevalence and impact of family violence underscores the importance of early detection and prevention. Addressing family violence requires prevention strategies across a range of contexts, such as social policy, culture, health care as well as the familial level [7]. Prevention of family violence has relied primarily upon education strategies, with a large emphasis on educating survivors of family violence [8], which have reported mixed results. For instance, Noughani and Mohtashami [9] found that dissemination of an educational booklet to women did not change the reported incident rate of family violence in their sample. However, a significant reduction in family violence after three 60-minute educational classes for women was reported by Taghdisi et al [10]. Education strategies have also targeted health professionals such as nurses [11]. Given the varying levels of success of these initiatives and the often-hidden nature of family violence, novel approaches for detecting family violence are needed.

Family violence increased during the COVID-19 pandemic, exacerbating the mental and physical health difficulties experienced by individuals, families, and communities [12]. Telehealth services and online mental health support tools proliferated during the COVID-19 pandemic [13,14]. The use of technology in psychology has been found to be useful across a range of settings. For instance, telehealth is effective in treating several mental health disorders [15] and previous research has examined the use of technology regarding family violence screening and interventions [16]. According to a review by El Morr and Layal [16], information and communication technologies facilitate greater levels of disclosure of family violence victimization than is achieved though in-person screening; however, only standardized screeners were studied and alternate means of disclosure (eg, free-text questions and voice signals) were not considered.

Progress in information technology, particularly artificial intelligence (AI) has led to some significant advancements in medical and public health interventions. In a recent systematic review, Qui et al [17] found that large AI models have been used in bioinformatics, medical diagnoses, imaging, informatics, education, robotics, and public health. These models include large language models, large vision models, and large multimodal models. The authors caution that large language models are not yet reliable and may generate information that can mislead users. Further challenges and risks identified by the review include bias, privacy issues, the resource intensive requirements associated with updating large models, and emphasize the importance of situating the models in line with human ethics.

Recent research has examined the use of machine learning in mental health, including screening for suicide risk [18], assessment of psychological distress [19], and detection of family violence in medical records [20,21] as well as using voice signal informed classification [22]. Voice signal data such as vocal pitch [23] and articulation [24] have been found to distinguish between the voice recordings of distressed and nondistressed individuals. Similar approaches using text-based analysis have been developed to identify and to detect reports of family violence on Twitter [25]. The analysis of social media content can provide valuable insights into risk factors, in particular textual markers of family violence. A similar approach has been taken where advanced AI models, such as transformer architecture have been applied to the analysis of annotated clinical notes to detect family violence [20]. In a review by Iyer and Meyer [18], timing patterns of speech were able to detect high risk of suicide callers compared with their comparison group with a median accuracy of 95%. Other vocal characteristics such as power spectral density sub-bands and mel-frequency cepstral coefficients demonstrated at least 80% accuracy in differentiating groups.

Machine learning and AI have also been used in predictive analytics across a range of settings, including crime and policing using Tweets [26] and mobile phone behavioral data [27] with high degrees of accuracy (70%-81%). Historically, predictive analysis regarding family violence has relied on police responses to questionnaires such as the Domestic Violence Safety Assessment Tool, and has poor predictive accuracy [28]. Given the success and accuracy previous AI tools have demonstrated at identifying psychological distress across both vocal and text-based settings, as well as predictive accuracy in some settings, research into predictive analytics and the use of AI to detect risk of family violence may have real world consequences, particularly in terms of prevention. However, to date, no research has amalgamated these findings to assess the accuracy of AI models in identifying the risk of family violence.

Detecting individuals who are at risk of perpetrating family violence is critical for the implementation of prevention strategies. The primary aim of this systematic review is to assess the accuracy of AI models in differentiating between individuals at risk of perpetrating family violence, versus those who are not, using textual or voice signal data. The following questions will inform this review: (1) What research using AI and machine learning has used textual or voice signal data to identify risk of family violence? and (2) What is the accuracy of such tools in differentiating between individuals at risk of perpetrating family violence versus those who are not?

## Methods

### Inclusion and Exclusion Criteria

An overview of inclusion and exclusion criteria is presented in Textbox 1.

**Inclusion and exclusion criteria.**

**Inclusion criteria**

- Include human participants.
- Involve adult participants.
- Use machine learning methods.
- Differentiate between low and high risk of family violence perpetration.
- Use voice signal data or linguistic (textual) data.
- Nonexperimental and experimental studies.
- Reporting metrics of classification accuracy.

**Exclusion criteria**

- Animal models.
- Child or adolescent perpetrators.
- Reviews (eg, narrative, systematic, meta-analysis, and meta-regression).
- Comments and editorials.
- The treatment of family violence.
- Involving child abuse.
- Involving elder abuse.
- The focus of enquiry is a condition other than family violence.

Additional inclusion criteria are that the papers are available in full text, and published in peer reviewed journals. Theses and dissertations will also be eligible for inclusion to yield the most exhaustive search possible. Languages other than English will be considered and translations conducted, subject to time and resource availability.

The decision to restrict the study population to adult participants is consistent with most research that recognizes adults as the key perpetrator population.

### Search Strategy

The search strategy and terms were informed by a preliminary search for relevant publications. The search will be restricted to the following databases: IEEE Xplore, PubMed, PsycINFO, EBSCOhost (Psychology and Behavioral Sciences Collection), and Computers and Applied Sciences Complete. ProQuest Dissertations and Theses A&I will also be used to search the grey literature. The full text will be searched using the following representative syntax (ie, Pubmed) (((“Domestic Violence“[Mesh] NOT (“Child Abuse”[Mesh] OR “Elder Abuse”[Mesh])) OR “family violence”[All Fields] OR “domestic violence”[All Fields] OR “domestic abuse”[All Fields] OR “intra-familial violence”[All Fields] OR “spousal violence”[All Fields] OR “spousal abuse”[All Fields] OR “interpersonal violence”[All Fields] OR “interpersonal abuse”[All Fields] OR “intimate partner violence”[All Fields]) AND ((“natural language processing”[Mesh] OR “natural language processing”[All Fields] OR “word embeddings”[All Fields]) OR (“signal processing, computer-assisted”[MeSH Terms] OR “speech analysis”[All Fields] OR “acoustic”[All Fields] OR “emotional speech”[All Fields] “voice”[All Fields] OR “MFCC”[All Fields]))) NOT “review”[publication type]. The reference lists of all articles included for review will be searched for any additional publications as well as relevant reviews.

### Study Selection

**Figure 1:**
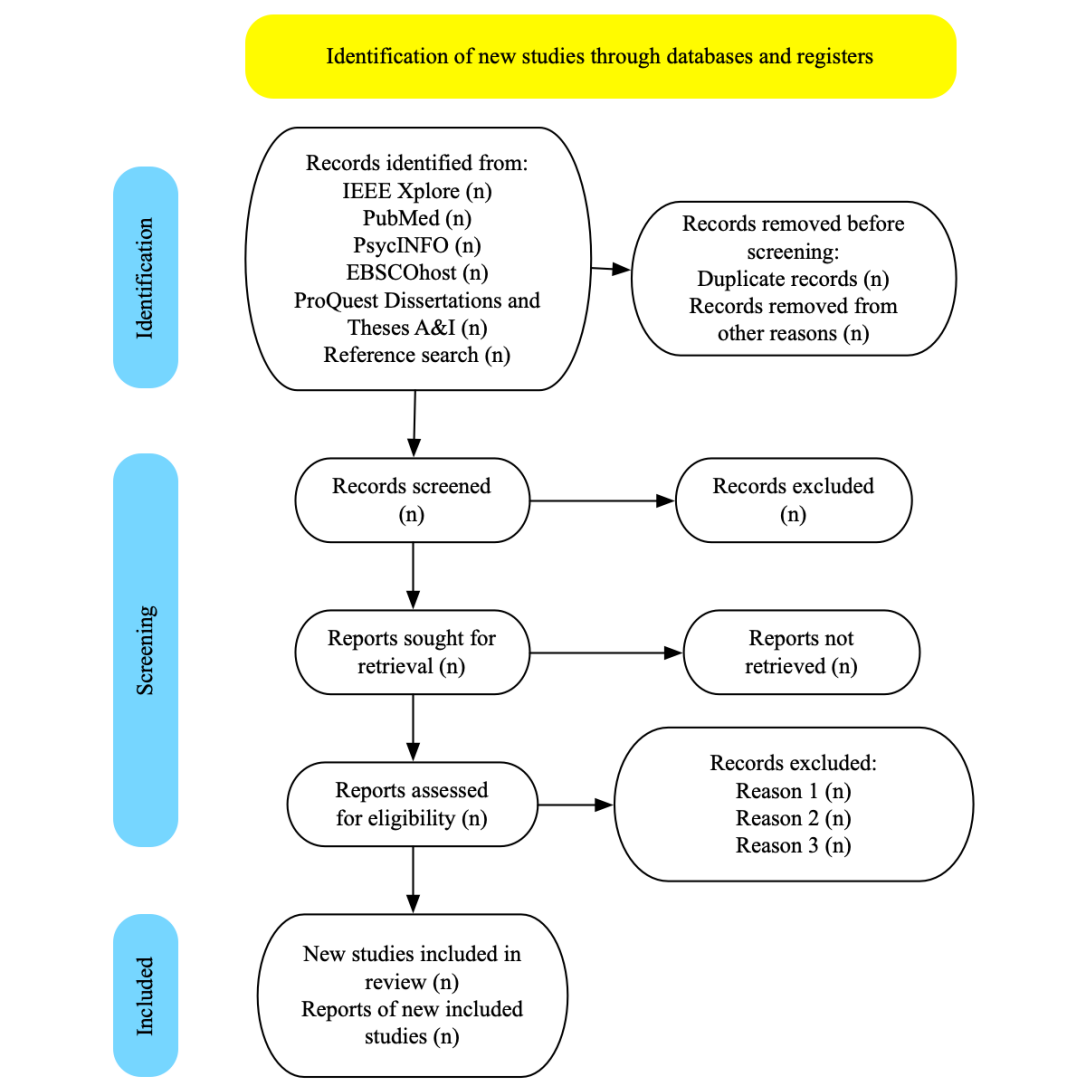
Overview of study selection. Searches are yet to be performed.

Retrieved studies will be loaded into NVivo (Lumivero) and title and abstract screening will be conducted by 2 researchers. A full-text review will be conducted by 2 authors and will resolve any discrepancies through discussion, or a third reviewer as needed. An overview of the study selection process is provided in Figure 1.

Studies that meet all inclusion criteria will be included in the review. An overview of the participant, intervention, comparator, outcomes, and time (PICOT) [29] is provided below (Textbox 2) and forms the basis of data extraction. Screening and reporting of included publications will be in accordance with the PRISMA (Preferred Reporting Items for Systematic Reviews and Meta-Analyses) guidelines (refer to Multimedia Appendix 1 for PRISMA checklist).

**Summary of the participant, intervention, comparator, outcomes, and time requirements.**

**Patient population**

- >18 years of age.

**Intervention of interest**

- Reviewing the use of text and voice signal-based machine learning tools used to predict risk of family violence.

**Comparison interventions**

- Not applicable.

**Primary outcomes**

- Accuracy of classification between low and high risk of family violence.

**Time (Year published)**

- 2000 and above.

**Other considerations**

- Language: all languages.
- Study designs: quantitative, experimental, and nonexperimental.
- Population: nonanimal investigations.

### Quality Assessment

Quality assessment will be performed by 2 researchers using the risk of bias tool in nonrandomized studies of interventions [30]. Included studies will be rated across the 7 domains including bias due to confounding, selection of participants, classification of interventions, deviations from intended interventions, missing data, measurement of outcomes (eg, reporting training versus test dataset accuracy), and selection of the reported result. A final rating of risk of bias will then be assigned to each included publication and across included publications as either low, moderate, serious, critical, or no information provided. This step is essential to ensure the validity and reliability of reported findings.

### Data Extraction

Data extraction will be conducted on NVivo and will be performed by 2 researchers, with regular meetings to discuss results. If there is disagreement regarding extraction, a third researcher will be involved in mediating. The following categories will be used to extract the data: study characteristics, participant characteristics, intervention and comparator characteristics, and outcome characteristics. Specific information will also be extracted relevant to vocal signal characteristics used, natural language processing methods, and machine learning methods.

### Synthesis Method

Initially, included studies will be synthesized using a narrative synthesis following guidelines proposed by Rodgers et al [31]. Data will be tabulated based on the information described above. The tabulated data will then be analyzed and clustered into groups based on various characteristics, such as population demographics and data type (eg, text or speech). Short summaries of each included paper will also be developed. Throughout the synthesis process, the research team will meet to critically evaluate the process and to resolve issues that may arise during the synthesis.

A random-effects meta-analysis will be conducted using the area under the receiver operating curve (AUC) and its SE, as reported in the included studies. Where an AUC value is not reported, a point estimate for the AUC will be obtained from the confusion matrix [32]. To be included in the analysis, the SE or CI must be reported. In the absence of SE values, these will be computed by the equivalence of the AUC to the Wilcoxon statistic or derived from reported CIs. If not provided, the corresponding authors will be contacted with a request to provide the necessary data. Separate random-effects meta-analyses will be performed by classification algorithm subgroup (eg, deep learning, gradient boosting random forest etc), where enough studies (>3) are available. Higgins *I*^2^ test will be used to evaluate the level of heterogeneity between included studies, with a level >56% indicating substantial levels of heterogeneity between included studies [33]. Publication bias will be ascertained through Egger regression and funnel plot analyses [34]. All analyses will be conducted using R software (version 4.2.0; R Foundation for Statistical Computing) and the “metafor” (ie, meta-analyses and funnel plots).

### Ethical Considerations

No participants will be involved in this review, as data will be extracted from existing published studies. Thus, ethics review is not required for this study. This review aims to contribute to a paucity of research in the areas of family violence risk prediction that uses textual and voice-signal based data. Findings from this review will be of particular importance internationally to health care providers such as telehealth services that leverage verbal cues to assess risk. Given the reported high incidence of family violence in the community, this will clarify the state of the literature, providing meaningful information that may be of interest to law enforcement, health care providers, and policy makers and may clarify the next steps needed to advance research in this area. In addition, this review will provide important information about the relevance of specific voice and text features in risk detection for the development of future algorithms.

## Results

As of October 2024, preliminary searches have not been conducted. Data will be extracted in line with the aims of this review. The results will include a narrative summary. The meta-analysis results will be presented using a forest plot. It is expected results will be published with the established protocol in a peer-reviewed journal.

## Discussion

The primary aim of this systematic review is to assess the accuracy of AI models in differentiating between individuals at risk of perpetrating family violence versus those who are not. Given that previous research has been accurate at identifying other forms of risk for example, suicide risk [18], and based on preliminary searches [22], it is anticipated that these models may report a high degree of accuracy in identifying individuals who are at high risk of perpetrating family violence.

Given that this is a nascent area of research, it is possible that there may be a high level of heterogeneity across the included studies, differences in the definitions used to identify family violence and there may be diversity in the vocal or textual characteristics used to discriminate high and low risk, as has been found in previous reviews [18]. Despite these potential limitations, the results of this review will clarify the state of the literature on the accuracy of machine learning models to identify individuals who are at high risk of perpetuating family violence. Importantly, they may contribute to the development of machine learning and AI models that can accurately predict individuals at risk of perpetrating family violence, thus contributing to the development of potential prevention and intervention strategies. In addition, the findings from this review will summarize what systems are involved in the detection of family violence.

Findings from this review may inform the development of surveillance models and contribute to evolving dialogue concerning ethical and political considerations on surveillance on domestic spaces, abusers co-opting smart speakers and the privatization response to family violence as outlined by Sparrow and colleagues [35].

Results from this literature review will be disseminated through academic journals and will likely be presented at academic conferences.

## Appendix

Multimedia Appendix 1 PRISMA (Preferred Reporting Items for Systematic analyses and Meta-Analyses) checklist.

## Abbreviations

AI: artificial intelligence
AUC: area under the receiver operating curve
PICOT: participant, intervention, comparator, outcomes and time
PRISMA: Preferred Reporting Items for Systematic Reviews and Meta-Analyses

## Data Availability

Data sharing is not applicable to this article as no datasets were generated or analyzed during this study.

## References

[1] Tolan P, Gorman-Smith D, Henry D (2006). Family violence. Annu Rev Psychol.

[2] Fulu E, Jewkes R, Roselli T, Garcia-Moreno C (2013). Prevalence of and factors associated with male perpetration of intimate partner violence: findings from the UN multi-country cross-sectional study on men and violence in Asia and the pacific. The Lancet Global Health.

[3] Lagdon S, Armour C, Stringer M (2014). Adult experience of mental health outcomes as a result of intimate partner violence victimisation: a systematic review. Eur J Psychotraumatol.

[4] Global Study on Homicide.

[5] Sardinha L, Maheu-Giroux M, Stöckl H, Meyer S, García-Moreno C (2022). Global, regional, and national prevalence estimates of physical or sexual, or both, intimate partner violence against women in 2018. The Lancet.

[6] (2023). Personal Safety, Australia.

[7] Regan PC, Durvasula RS (2015). A brief review of intimate partner violence in the United States: nature, correlates, and proposed preventative measures. Interpersona.

[8] Sinnott T, Artz S (2016). A literature review of strategies for the prevention of intimate partner violence during the childbearing years. Int J Child Youth Family Stud.

[9] Noughani F, Mohtashami J (2011). Effect of education on prevention of domestic violence against women. Iran J Psychiatry.

[10] Taghdisi MH, Estebsari F, Dastoorpour M, Jamshidi E, Jamalzadeh F, Latifi M (2014). The impact of educational intervention based on empowerment model in preventing violence against women. Iran Red Crescent Med J.

[11] Khumisi ET, De WM, Van WNC (2015). Educating nurses on intervention in and prevention of intimate partner violence: a systematic review. Afr J Phys Health Educ Recreat Dance.

[12] Kourti A, Stavridou A, Panagouli E, Psaltopoulou T, Spiliopoulou C, Tsolia M, Sergentanis TN, Tsitsika A (2023). Domestic violence during the COVID-19 pandemic: a systematic review. Trauma Violence Abuse.

[13] Koonin LM, Hoots B, Tsang CA, Leroy Z, Farris K, Jolly T, Antall P, McCabe B, Zelis CBR, Tong I, Harris AM (2020). Trends in the use of telehealth during the emergence of the COVID-19 pandemic - United States, january-march 2020. MMWR Morb Mortal Wkly Rep.

[14] Sistani F, Rodriguez de Bittner M, Shaya FT (2022). COVID-19 pandemic and telemental health policy reforms. Curr Med Res Opin.

[15] Thomas N, McDonald C, de Boer K, Brand RM, Nedeljkovic M, Seabrook L (2021). Review of the current empirical literature on using videoconferencing to deliver individual psychotherapies to adults with mental health problems. Psychol Psychother.

[16] El Morr C, Layal M (2020). Effectiveness of ICT-based intimate partner violence interventions: a systematic review. BMC Public Health.

[17] Qiu J, Li L, Sun J, Peng J, Shi P, Zhang R, Dong Y, Lam K, Lo FP, Xiao B, Yuan W, Wang N, Xu D, Lo B (2023). Large aI models in health informatics: applications, challenges, and the future. IEEE J Biomed Health Inform.

[18] Iyer R, Meyer D (2022). Detection of suicide risk using vocal characteristics: systematic review. JMIR Biomed Eng.

[19] Iyer R, Nedeljkovic M, Meyer D (2022). Using vocal characteristics to classify psychological distress in adult helpline callers: retrospective observational study. JMIR Form Res.

[20] Botelle R, Bhavsar V, Kadra-Scalzo G, Mascio A, Williams MV, Roberts A, Velupillai S, Stewart R (2022). Can natural language processing models extract and classify instances of interpersonal violence in mental healthcare electronic records: an applied evaluative study. BMJ Open.

[21] Tabaie A, Zeidan AJ, Evans DP, Smith RN, Kamaleswaran R (2022). A novel technique to identify intimate partner violence in a hospital setting. West J Emerg Med.

[22] Muraleedharan A, Garcia-Constantino M (2022). Domestic violence detection using smart microphones. https://link.springer.com/chapter/10.1007/978-3-031-21333-5_36.

[23] Kandsberger J, Rogers SN, Zhou Y, Humphris G (2016). Using fundamental frequency of cancer survivors' speech to investigate emotional distress in out-patient visits. Patient Educ Couns.

[24] Scherer S, Morency LP, Gratch J, Pestian J (2015). Reduced vowel space is a robust indicator of psychological distress: a cross-corpus analysis. https://doi.org/10.1109/ICASSP.2015.7178880.

[25] Al-Garadi MA, Kim S, Guo Y, Warren E, Yang YC, Lakamana S, Sarker A (2022). Natural language model for automatic identification of intimate partner violence reports from Twitter. Array (N Y).

[26] Gerber MS (2014). Predicting crime using Twitter and kernel density estimation. Decision Support Systems.

[27] Bogomolov A, Lepri B, Staiano J, Oliver N, Pianesi F, Pentland A (2014). Once upon a crime: towards crime prediction from demographics mobile data. https://dl.acm.org/doi/proceedings/10.1145/2663204.

[28] Ringland C (2018). Domestic violence safety assessment tool (DVSAT) and intimate partner repeat victimisation, the bureau of crime statistics and research. NSW Crime Just Bull.

[29] Riva JJ, Malik KM, Burnie SJ, Endicott AR, Busse JW (2012). What is your research question? An introduction to the PICOT format for clinicians. J Can Chiropr Assoc.

[30] Sterne JA, Hernán MA, Reeves BC, Savović J, Berkman ND, Viswanathan M, Henry D, Altman DG, Ansari MT, Boutron I, Carpenter JR, Chan A, Churchill R, Deeks JJ, Hróbjartsson A, Kirkham J, Jüni P, Loke YK, Pigott TD, Ramsay CR, Regidor D, Rothstein HR, Sandhu L, Santaguida PL, Schünemann HJ, Shea B, Shrier I, Tugwell P, Turner L, Valentine JC, Waddington H, Waters E, Wells GA, Whiting PF, Higgins JP (2016). ROBINS-I: a tool for assessing risk of bias in non-randomised studies of interventions. BMJ.

[31] Rodgers M, Sowden A, Petticrew M, Arai L, Roberts H, Britten N, Popay J (2009). Testing methodological guidance on the conduct of narrative synthesis in systematic reviews: effectiveness of interventions to promote smoke alarm ownership and function. Evaluation.

[32] Sokolova M, Lapalme G (2009). A systematic analysis of performance measures for classification tasks. Information processing & management.

[33] Higgins JPT, Thompson SG (2002). Quantifying heterogeneity in a meta-analysis. Stat Med.

[34] Sterne JAC, Sutton AJ, Ioannidis JPA, Terrin N, Jones DR, Lau J, Carpenter J, Rücker G, Harbord RM, Schmid CH, Tetzlaff J, Deeks JJ, Peters J, Macaskill P, Schwarzer G, Duval S, Altman DG, Moher D, Higgins JPT (2011). Recommendations for examining and interpreting funnel plot asymmetry in meta-analyses of randomised controlled trials. BMJ.

[35] Sparrow R, Andrejevic M, Harris B (2023). Should we embrace “Big Sister”? Smart speakers as a means to combat intimate partner violence. Ethics Inf Technol.

